# Acute renal failure due to rhabdomyolysis following dengue viral infection: a case report

**DOI:** 10.1186/1752-1947-7-195

**Published:** 2013-07-26

**Authors:** Aruna Wijesinghe, Nanthini Gnanapragash, Gayan Ranasinghe, Murugapillai K Ragunathan

**Affiliations:** 1 Ward 45, National Hospital of Sri Lanka, Colombo, Sri Lanka

**Keywords:** Dengue fever, Myositis, Rhabdomyolysis, Acute renal failure

## Abstract

**Introduction:**

With more than one-third of the world’s population living in areas at risk for transmission, dengue fever is a leading cause of illness and death in the tropics and subtropics. Despite the high incidence of dengue fever, rhabdomyolysis leading to acute renal failure is an extremely rare complication of dengue fever. Only a few such cases have been reported in the literature.

**Case presentation:**

We describe the case of a 42-year-old, previously healthy Sri Lankan Sinhalese man who developed acute renal failure due to rhabdomyolysis following dengue virus infection. He was transferred to our institution with a five-day history of fever, headache, myalgia, impaired level of consciousness, and reduced urinary output. He was hemodynamically stable and did not have evidence of plasma leakage. His serology for dengue immunoglobulin M and immunoglobulin G was positive, and biochemical investigations disclosed evidence of rhabdomyolysis and acute renal failure. He was treated with induced alkaline diuresis and hemodialysis, and he experienced an uncomplicated recovery.

**Conclusion:**

The occurrence of acute renal failure significantly increases the mortality of patients with dengue fever. Therefore, early diagnosis and early management are crucial in rhabdomyolysis complicating dengue fever to prevent established acute renal failure. It should be kept in mind that the threshold for suspecting rhabdomyolysis is very low in dengue fever. Creatinine phosphokinase levels should routinely be measured in all patients with severe dengue fever for early detection of rhabdomyolysis to prevent acute renal failure.

## Introduction

With more than one-third of the world’s population living in areas at risk for transmission, dengue fever is a leading cause of illness and death in the tropics and subtropics. Dengue fever is caused by any of the four dengue virus serotypes (DEN 1–4), which are closely related. Dengue virus serotypes 2 and 3 were the predominant circulating serotypes in Sri Lanka until 2009. However, since 2010, dengue serotype 1 has become the predominant serotype in Sri Lanka, accounting for more than 95% of dengue infections [1]. Acute renal failure (ARF) is a rare but well-recognized complication of dengue fever, with an incidence which varies from 0.3% to 3.3% in different populations [2-4]. It is almost always associated with hypotension and hypoperfusion secondary to the severe form of dengue hemorrhagic fever and dengue shock syndrome [3,4]. ARF due to rhabdomyolysis is an extremely rare complication of dengue fever, and only a few such cases have been reported in the world [5-7]. We describe the case of a 42-year-old Sri Lankan man who developed ARF due to rhabdomyolysis following dengue virus infection.

## Case presentation

A 42-year-old, previously healthy Sri Lankan Sinhalese man was transferred to our institution with a five-day history of fever, headache, myalgia, impaired level of consciousness, and reduced urinary output. Upon admission, he was febrile, drowsy (Glasgow Coma Scale score 14/15) and icteric, with blood pressure of 140/90mmHg, pulse of 96 beats/min, capillary refilling time of <2 seconds, breathing frequency of 16/min, and oxygen saturation of 97% on room air. He did not have evidence of pleural effusion or ascites. Laboratory investigations upon admission showed thrombocytopenia, leukopenia, and increased levels of liver transaminases, serum bilirubin, prothrombin time, serum ammonia, blood urea, serum creatinine, and creatinine phosphokinase (CPK). Dipstick urinalysis was strongly positive for blood, and myoglobinuria was confirmed by enzyme-linked immunosorbent assay on a random urine sample. Both immunoglobulin G (IgG) and immunoglobulin M (IgM) dengue antibody tests were positive, suggesting acute dengue infection. The serological test for leptospirosis was negative. The investigation results are summarized in Table 1. The patient was treated with intravenous bicarbonate and eventually required hemodialysis due to oliguria and severe metabolic acidosis. He was discharged after nine days with a normal platelet count and CPK. His follow-up was uneventful.

**Table 1:** Laboratory investigations of case presentation

**Days from admission**	**Day 1**	**Day 3**	**Day 5**	**Day 7**	**Reference value**
Hemoglobin g/L	15.2	14.6	14.8	15.4	13 to 16
Platelets (×10^9^/L)	38	32	96	144	150 to 450
White blood cells (×10^9^/L)	2,100	4,700	6,400	6,600	4000 to 10,000
CPK (U/L)	6,240	4,880	2,260	1,840	25 to 174
Serum creatinine (μmol/L)	6.3	3.2	2.6	2.1	0.6 to 1.2
Blood urea (mmol/L)	32.4	21.2	18.4	14.3	2.9 to 8.2
Sodium (mmol/L)	136	132	140	138	135 to 148
Potassium (mmol/L)	5.8	5.2	4.9	4.8	3.5 to 5.1
AST (U/L)	3,120	2,080	1,280	846	10 to 35
ALT (U/L)	1,270	850	570	325	10 to 40
Serum bilirubin (μmol/L)	38	44	–	32	5.1 to 22
International Normalized Ratio	2.1	1.6	1.4	1.1	1 to 1.3

*CPK* creatinine phosphokinase, *AST* aspartate aminotransferase, *ALT* alanine aminotransferase.

## Discussion

The occurrence of ARF secondary to rhabdomyolysis associated with dengue infection is extremely rare. To the best of our knowledge, only a few cases have been reported previously [5-7]. The exact pathogenesis of myositis and consequent rhabdomyolysis in dengue infection is not clear, and two possible mechanisms have been postulated: direct viral invasion of muscle and immune-mediated damage of muscle fibers. *In vitro* studies have shown that the human muscle satellite cells have a high affinity for the dengue virus and a high efficiency infection and replication of the dengue virus in the human primary skeletal muscle cells, strengthening the theory of direct muscle invasion by dengue virus [8,9]. On the other hand, as direct invasion of muscle by virus has not been demonstrated consistently, a more likely cause of myositis and rhabdomyolysis in dengue fever could be the release of myotoxic cytokines, particularly the tumor necrosis factor [10,11]. Dengue virus infection has been shown to increase the production of the tumor necrosis factor in humans [12].

Although myoglobin is not nephrotoxic per se, it is the true pathogenic factor in rhabdomyolysis-induced acute kidney injury [13]. The exact mechanisms by which rhabdomyolysis impairs the glomerular filtration rate are unclear, but experimental evidence suggests that intrarenal vasoconstriction, direct and ischemic tubular injury, and tubular obstruction might play a role [13].

As our patient did not have any history suggestive of other well-known causes of rhabdomyolysis, such as strenuous exercise, drug use, electrolyte imbalances, the co-occurrence of ARF with peak CPK levels, and myoglobinuria make the diagnosis of ARF due to rhabdomyolysis following dengue virus infection more probable.

It is important to note that, in our patient, ARF was associated with relatively low CPK levels. Although there is no defined threshold value of serum CPK above which the risk of acute kidney injury is markedly increased, the risk of acute kidney injury in rhabdomyolysis is usually low when CPK levels are less than 15,000 to 20,000U/L [14]. Acute kidney injury has been reported with CPK values as low as 5,000U/L, especially when co-existing conditions such as sepsis, dehydration, and acidosis are present [15].

A recent study in adults found that dengue fever complicated by ARF has a mortality rate of 60% [4]. Therefore, early diagnosis and early management are crucial in rhabdomyolysis to prevent or to minimize established ARF. Early diagnosis of rhabdomyolysis requires demonstration of elevated levels of serum CPK and urinary myoglobin. Although several methods are available for the detection of urinary myoglobin, accurate quantitative tests for myoglobin in urine remain difficult to achieve [16]. The dipstick method was developed to measure blood in the urine but does not differentiate hemoglobin, myoglobin, and red blood cells. The addition of a separation step to remove hemoglobin allows the preferential detection of myoglobin over hemoglobin, but this technique yields both false-positive and false-negative results [17]. Immunoassays, which are validated for serum myoglobin measurement, can be used, with suitable modifications, to measure urinary myoglobin [17]. Serum myoglobin levels peak well before serum CPK levels, but, given its fast clearance, it has low sensitivity for the diagnosis of rhabdomyolysis [13]. Hence, serum myoglobin level was not measured in our patient.

The mainstay of the management of rhabdomyolysis, whether it is associated with ARF or not, is early, aggressive repletion of fluids, with the amount administered being dependent on the severity of the rhabdomyolysis, the degree of renal impairment, and the volume status of the patient [13].

On the other hand, the mainstay of management of dengue fever is to give just enough fluid to maintain adequate circulation while being careful not to deliver more than is absolutely necessary––a very delicate balancing act to prevent fluid overload. Aggressive parenteral fluid therapy in a dengue patient can result in fluid overload and worsening of plasma leakage, leading to increased morbidity and mortality [18]. To limit the risk of the development of fluid overload, parenteral fluid therapy should be kept to the minimum required to maintain cardiovascular stability until permeability reverts to a normal level [19]. CPK levels should routinely be measured in all patients with severe dengue fever for early detection of rhabdomyolysis to minimize ARF.

## Conclusion

The occurrence of ARF significantly increases the mortality of patients with dengue fever. Therefore, early diagnosis and early management are crucial in rhabdomyolysis complicating dengue fever to prevent established ARF. As our patient had relatively low levels of CPK with the onset of rhabdomyolysis, it should be kept in mind that the threshold for suspecting rhabdomyolysis is very low in dengue fever. CPK levels should routinely be measured in all patients with severe dengue fever to prevent ARF due to rhabdomyolysis.

## Consent

Written informed consent was obtained from the patient for publication of this case report and any accompanying images. A copy of the written consent is available for review by the Editor-in-Chief of this journal.

## Competing interests

The authors declare that they have no competing interests.

## Authors’ contributions

Analysis and interpretation of patient data and literature review were done by AW, NG, and GR. MKR guided the other authors in reporting this case and corrected the final manuscript. All authors were involved in the management of the patient and read and approved the final manuscript.

## Authors’ information

AW (MBBS) is a registrar in medicine. NG (MBBS, MD) is a senior registrar in medicine. GR (MBBS, MD) is a senior registrar in medicine. MKR (MBBS, MD, FRCP) is a senior consultant physician.
